# Food Insecurity and Mental Distress among Mothers in Rural Tigray and SNNP Regions, Ethiopia

**DOI:** 10.1155/2019/7458341

**Published:** 2019-06-19

**Authors:** Tarik Taye Birhanu, Amare Worku Tadesse

**Affiliations:** Addis Continental Institute of Public Health, Ethiopia

## Abstract

Access to safe and adequate food is a basic human right under Article 25 of the Universal Declaration of Human Rights. Globally, more than 870 million people consume less calories than they require, which can lead to disabling physical and mental health outcomes. This study was designed to investigate the association between household food insecurity and mental distress among mothers in the Tigray and SNNP regions of Ethiopia. A community based cross-sectional survey was completed on a total of 2,992 households. A linear multiple regression model was used to study the association between food insecurity and mental distress. More than half of the study participants, 57.9%, were experiencing food insecurity. The prevalence of mental distress among the mothers was 39%. Food insecurity was significantly associated with mental distress after controlling for socioeconomic covariates. Integrating screening and management of mental distress would result in a better health status of the mothers and those under their care.

## 1. Introduction

Due to the emerging global changes in social, economic, political, and climatic conditions, food insecurity (FI) is becoming a focus of public health and political attention in low and middle income countries, particularly in sub Saharan Africa (SSA) [1]. According to the Food and Agriculture Organization (FAO) of the United Nations, food insecurity is defined as an insecure access to sufficient food for a healthy and active life [2]. Access to safe and adequate food is a basic human right under Article 25 of the Universal Declaration of Human Rights. Globally, more than 870 million people consume less calories than they require, which can cause disabling outcomes by affecting the physical and mental health of those affected [3].

The number of people experiencing food insecurity is expected to double by the year 2020 [4]. Around one third of the global population, and more than 70% of the African urban population, live in slums, with a projection of 1.7 billion people living in a similar way by 2030, compounded by a substantial rate of internal and external migration, where access to basic food could be problematic [5]. In a longitudinal survey of African American and white welfare recipient women from the United States, one third of the respondents experienced food insecurity [6]. Findings from a cross-sectional study in two socioeconomically deprived South African districts showed that 60% of the participants were suffering from severe food insecurity [7]. In Ethiopia, a comparative cross-sectional study in East and West Gojjam revealed a 55.3% prevalence of food insecurity among the surveyed households [8].

Food insecurity was found to be associated with adverse health outcomes among those affected [9]. Even though all members of the household suffer from the effects of food insecurity, women and children are the ones affected the most [10]. A compromise in maternal capacity to provide adequate care to the child contributes to child malnutrition which in sum contributes to one third of under-five deaths in developing countries [11]. Hence, the impact of maternal food insecurity is transgenerational [12]. Also, having a child with a failure to thrive could undermine a mother's well-being through increased mental distress because of the extra effort required to take care of her child and the pressure from overt disappointment and criticism expressed by close relatives [13].

Rates of food insecurity vary considerably by race, gender, and socioeconomic status and other variables [6]. In addition to the compelling theoretical foundation which indicates that food insecurity is directly related to mental health morbidities [14], multiple epidemiological studies have shown that there is a link between food insecurity and mental distress especially in low and middle income countries [1].

Mental distress represents a constellation of subjective complaints characterized by symptoms of sleeplessness, exhaustion, irritability, poor memory, difficulty in concentrating, and other medically unexplained physical symptoms [12]. Mental distress is one of the leading causes of morbidity among women of reproductive age, with an overwhelmingly greater burden in sub-Saharan Africa where the socioeconomic and political situation is often unstable [7].

Data from 17 countries revealed a 12% to 47% lifetime rate of mental distress [5]. A cross-sectional study conducted among mothers in Rio de Janeiro, Brazil, 6 weeks after giving birth revealed a 40% prevalence of common mental disorders (CMD) and a 24% prevalence of severe mental disorders [11]. Additional findings from a cross-sectional study in Goba and Robe town of Bale Zone, South Eastern Ethiopia, revealed that one in three women has a significant mental health problem [15]. A higher score of FI is associated with greater odds of any past-year mental distress [odds ratio (OR), 1.2]. Moreover, food insecurity is significantly associated with meeting the screening criteria for major depressive disorders (OR 1.56-4.75). Food insecurity is a mirror of low socioeconomic status, although it is not clear if food insecurity leads to mental distress or if mental distress drives food insecurity [9]. Comorbid physical health problems are major causes of premature death in people with mental distress, associated with a 60% increased mortality [5]. Furthermore, women living in persistent food-insecure households were 2.85 times more likely to experience mental distress compared to those who were living in a food secure household [4, 16]. Similar findings from a community based cross-sectional study conducted in Jimma Zone, South West Ethiopia, also revealed higher rates of depression, 62%, and anxiety, 65%, in food-insecure households when compared to food-secure households (27% and 34%, respectively) [4].

The impact of food insecurity on the physical health is well established; however, limited empirical studies are available in the area of association between household food insecurity and mental distress among mothers, particularly in Ethiopia. Hence, this study was designed to investigate the association of household food insecurity and mental distress from a baseline study of mothers as part of the evaluation process of the “Alive and Thrive project” on child undernutrition. Since food insecurity brings a substantial public mental distress and the impact is potentially modifiable [7] results from this study could be used in designing, monitoring, and evaluating food security and mental health programs and would also assist in building a body of knowledge which eventually could influence public health policy in Ethiopia.

## 2. Materials and Methods

Ethiopia is a federal democratic republic composed of 11 regions: nine regional states and two city administrations, with more than 84% of the population living in the rural areas of the country [17]. This study was conducted in rural parts of Tigray and SNNP regions. Eighty nine program operational woredas (35 from Tigray and 54 from SNNP region) of the Integrated Family Health Program (IFHP) were selected as study sites. From the 89 woredas 75 enumeration areas from 56 woredas were selected using a formal list and map designed by the Central Statistical Authority (CSA) used in the 2005 Ethiopian Demographic and Health survey (EDHS 2005).

A community based cross-sectional survey was used to study the magnitude of food insecurity and mental distress among women of SNNP & Tigray regions and to assess the association between the two variables. The study population consisted of women who lived in the rural Tigray and SNNP regions between June and September, 2010, with a child under 5 years of age.

The study objectives are to measure the magnitude of food insecurity among mothers who were beneficiaries of the Alive and Thrive project in rural Ethiopia, to measure the magnitude of mental distress and to assess the association and direction of association between food insecurity and mental distress. Sample size was calculated for the three study objectives using Epi info version 7. The assumptions were Type-I error of 5%, Power of 80%, and Design Effect of 2. Moreover, the prevalence of food insecurity as 55.3% [8] and mental distress as 30% [15] and the combination of these two types of prevalence were considered to test for the association as indicated in the third objective of the study. Hence, the calculation resulted in a sample size, 1575. However, for practical purposes, a total sample size of 2,992 available from the existing data set was used for the study.

Samples were selected using a two-stage cluster sampling method. First, the two regions were divided into enumeration areas (EAs) which consisted of about 150 to 200 households according to the original assignment by the Central Statistics Authority (CSA) for the Demographic and Health Survey (DHS). Out of the list of all EAs, 75 EAs were selected using a lottery method. These selected 75 EAs were the primary sampling units, and the specific sample size of households (HHs) in each EA was allocated using the Probability Proportional to Size (PPS). The secondary sampling units, HHs, were selected using systematic random sampling method for each EA. A complete listing of all households in the study area was done and separate sampling was prepared for all households with children under 5 years of age. Using the sampling frame, households were randomly selected to participate in the study from the 75 enumeration areas (EAs).

### 2.1. Measures

The data was collected by trained data collectors using a structured three-part questionnaire. Part one contained 21 items that describe the identification and sociodemographic status of participants. The second part dealt with household food insecurity. Food insecurity was measured using “Household Food Insecurity Access Scale “(HFIAS),” version 3, a standardized check list which measures HH food insecurity using 9 items with a 30-day recall period developed using results frameworks by the Food and Nutrition Technical Assistance (FANTA) Project of the United States Agency for International Development (USAID) [3]. The tool was first translated to Amharic (local language) and back translated to English to check for language consistency. Then, all tools were pretested in a location similar to the study area to check for language clarity. Participants were interviewed by a trained data collector at the household level. The nine questions focused on the mother's or caretaker's experience on an occurrence related to food insecurity with a “Yes” or “No” response. If the mother or caretaker replied Yes to any of the occurrence questions, the data collector would further ask a frequency-of-occurrence question with three scales labelled Rarely, Sometimes, and Often [18]. If the mother or caretaker replied “No” to any of the occurrence questions, the administrator would jump to subsequent occurrence questions.

Mental distress was measured using a self-reporting questionnaire (SRQ-20) which was originally developed by the World Health Organization (WHO) to screen for mental health problems in primary health care [19]. The tool was validated in several subsequent studies done in Ethiopia through multiple parameters of validity, such as face validity, content validity, criterion validity, and construct validity, and also proved to have an acceptable level of inter- rater and internal item reliability [20–22]. Internal item consistency was measured for this particular study and it yielded a Cronbach's alpha of 0.876.

For quality assurance purposes, data was encoded with a double entry process using two data encoders simultaneously.

The data set was checked for completeness and was cleaned using SPSS Version 23.0. Food insecurity was considered as the exposure variable of interest and mental distress was computed as the outcome variable.

The sociodemographic and economic status of study participants such as age of the mother, marital status, educational status, household head, having additional job aside from household chores, history of employment 12 months prior to data collection, respondent's main occupation, remuneration for main occupation and location of work (at home or outside of home), household construction materials, source of drinking water, type of toilet facilities, and usable assets possession were measured as independent variables. These variables were run together with respondent's status of food insecurity using multiple linear regression by entering all independent variables in the model. Finally, these variables were controlled to test for a possible linear relationship between the exposure variable (food insecurity) and the outcome variable (mental distress).

We have used the 4 scale categories for level of FI as food secure, mild food insecurity, moderate food insecurity, and sever food insecurity. [3].

The outcome variable, mental distress, was measured with a self-reporting questionnaire (SRQ-20) which contains 20 items that were scored as “0” or “1” depending on the absence and presence of symptoms, yielding a maximum score of 20 and a minimum of 0. The prevalence of mental distress was decided using a score of “7” [19]. Mothers or caretakers who scored below 7 were classified as experiencing no mental distress and those who scored 7 and above were classified as experiencing mental distress.

The research proposal was submitted to the Ethical Review Board of Addis Continental Institute of Public Health (ACIPH). Following ethical clearance, an official letter from the Ethical Review Board was submitted to the Public Health Nutrition Department of ACIPH along with an application to access the available data set.

## 3. Results and Discussion

### 3.1. Results

A total of 2,992 mothers were enrolled in the study from the 75 EAs in the two regions. Half of the respondents were between the age of 25 and 34 (Table 1). Most (97.5%) have no history of employment in the past 12 months (see Table 1). Moreover, highest proportion of mothers reported that they are married (91.5%) (see Table 1). Regarding the highest educational status attained by the study participants, majority (66.4%) of them reported that they have not attended any formal education (see Table 1). Furthermore, 93.1% of the mothers reported that someone other than the mother is the head of the household (see Table 1).

Household food insecurity was assessed using the responses of the 2,992 study participants as representatives of their respective households. The items were tested for consistency and resulted in a Cronbach's alpha score of 0.876. According to the results obtained from the study, one third of the households were food secure (34.4%). However, 36.1% of the households were moderately food insecure and severe food insecurity was present in 21.8% of the households. The remaining households (7.7%) were experiencing mild food insecurity.

The study participants were also assessed for mental distress for 30 days preceding the survey. Mothers scoring less than 7 on the standard mental distress measuring scale (SRQ-20) were classified as not experiencing mental distress and mothers scoring 7 and above on the scale were classified as experiencing mental distress. A substantial proportion of the mothers (39%) were experiencing mental distress during the 30 days preceding the survey.

In order to assess the association between food insecurity and mental distress, a multiple regression analysis was employed by entering all independent variables using SPSS v.23.0 to control for effects of age, history of employment, main occupation, means of remuneration, location of work of the mother, marital status of the mother, maternal education status, HH head type, HH construction materials, main source of drinking water, toilet facility, and number of usable assets. Characteristics of the independent variables were entered into the model separately to observe the relationship among different covariates while running the model.

Results of the multiple linear regression revealed that mental distress is significantly associated with household food insecurity (R2 adjusted = 10.6%, P value ≤ 0.001) (see Table 2). According to the results, 10.6% of the changes in the mental distress score is attributed to the status of food security. Furthermore, the multiple linear regression revealed that a 1 score increase on the household food insecurity scale was associated with a 0.297 increase on the mental distress scale with a constant SRQ-20 mental distress score of 11.57 while keeping all the other independent variables constant.

## 4. Discussion

The findings revealed that more than half (57.9%) of the households studied were experiencing moderate to severe food insecurity. A cross-sectional study conducted in East and West Gojjam, Ethiopia, resulted in a food insecurity prevalence of 55.3%, which is parallel with what was found in this study. The survey revealed that about 39% of the study participants were mentally distressed. The results resemble other similar studies conducted elsewhere. A data from 17 countries similarly showed a prevalence of mental distress between 12% and 47%. [5]. In a similar way, a cross-sectional study conducted in Goba and Robe town of Bale zone, SNNPR, Ethiopia, resulted in a 33.3% prevalence of mental distress [15]. The aforementioned studies confirm that the prevalence of food insecurity and mental distress follow a similar pattern in different parts of Ethiopia.

According to the findings, food insecurity and mental distress are directly and significantly associated. A one increase on the food insecurity score would bring a 0.209 increase on the mental distress score. Despite methodological differences, the results are comparable with similar studies from other parts of Africa. A hospital based cohort study conducted in the peri-urban Ghana among people living with HIV revealed that people living in persistent food insecure conditions are 2.85 times more likely to experience mental distress compared to those who are living in a food secure situation [16]. Despite the similarities in the direction of association, the study in Ghana showed a relatively strong association compared to this study. This dissimilarity might be due to the institutionalization of the study from Ghana and the fact that participants were on followup for HIV as well.

The strength of this study includes its large sample size in two separate regions of the country. This would give a good power and representativeness to the mothers who live in Tigray and SNNP regions. Some of the limitations of the study are its focus only on rural women and its lack of generalizability to the whole country since the data is representative of only two regions out of 9 regions and 2 city administrations. Moreover, the data set is six years old and it might not be ideal to describe the current situation of food insecurity and mental distress in the respective area.

## 5. Conclusions

Based on findings from this study, over 57.9% of households of mothers living in Tigray and SNNP regions were experiencing food insecurity. The prevalence of mental distress among mothers in these two regions was recorded to be substantial (39%). Food insecurity was found to be significantly associated with mental distress.

## Figures and Tables

**Table 1:** Socio-economic characteristics of the study participants in SNNP and Tigray regions, 2010.

Variables		n	%
Age			

	15-19	120	4
	20-24	569	19
	25-29	879	29.4
	30-34	652	21.8
	35-39	536	17.9
	≥40	236	7.9

Additional job aside from household chores			

	Yes	837	28.2
	No	2134	71.8

History of employment in the past 12 months			

	Yes	73	2.5
	No	2901	97.5

Mother's main occupation			

	Farmer or family work	1289	43.1
	House wife	1495	50
	Other occupation	208	7

Remuneration for main occupation			

	Money	138	4.6
	In-kind	76	2.6
	Money and in-kind	38	1.3
	Nothing	2777	91.5

Location of work			

	Home	1570	52.6
	Away from home	616	20.7
	Both at home and away from home	797	26.7

Marital status of the mother			

	Single	41	1.4
	Married	2737	91.5
	Widowed	56	1.9
	Divorced	105	3.5
	Separated	48	1.6
	Married more than one spouse	2	0.1

Maternal educational status			

	No formal education	1986	66.4
	Grade 1-6	798	26.7
	Grade 7-12	195	6.5
	Higher education	13	0.4

Head of the household			

	Mother	203	6.9
	Other than the mother	2785	93.1

Main source of drinking water for the household			

	Piped water	1202	40.2
	Non-piped water	1790	59.8

Toilet facility			

	Traditional pit latrine	2382	79.6
	Ventilated improved pit (VIP) latrine	49	1.6
	No facility/Bush/Field	555	18.8

Number of functional asset possession			

	Lowest tertile	1016	34%
	Middle tertile	996	33.30%
	Highest tertile	975	32.70%

Household construction materials			

	Traditional construction materials	2953	98.7
	Modern construction materials	39	1.3

**Table 2:** Results from multivariable linear regression showing the association between food insecurity and mental distress.

Model	Unstandardized Coefficients		Standardized Coefficients	t	Sig.	95.0% CI for B	
	B	SE	B			Lower	Upper
(Constant)	11.570	2.384		4.852	0.001	6.894	16.245

HFIAS	.209	.013	.297	16.414	0.001	.184	.234

*∗* Adjusted for maternal marital status, age, education and SES

## Data Availability

The data used to support the findings of this study are available from the corresponding author upon request.

## References

[1] Weaver L. J., Hadley C. (2009). Moving beyond hunger and nutrition: a systematic review of the evidence linking food insecurity and mental health in developing countries. *Ecology of Food and Nutrition*.

[2] Maes K. C., Hadley C., Tesfaye F., Shifferaw S. (2010). Food insecurity and mental health: Surprising trends among community health volunteers in Addis Ababa, Ethiopia during the 2008 food crisis. *Social Science & Medicine*.

[3] Jones A. D., Ngure F. M., Pelto G., Young S. L. (2013). What are we assessing when we measure food security? A compendium and review of current metrics. *Advances in Nutrition*.

[4] Hadley C., Tegegn A., Tessema F., Cowan J. A., Asefa M., Galea S. (2008). Food insecurity, stressful life events and symptoms of anxietry and depression in east Africa: evidence from the Gilgel Gibe growth and development study. *Journal of Epidemiology and Community Health*.

[5] Jenkins R., Baingana F., Ahmad R., McDaid D., Atun R. (2011). Mental health and the global agenda: core conceptual issues. *Mental Health in Family Medicine*.

[6] Siefert K., Heflin C. M., Corcoran M. E., Williams D. R. (2004). Food insufficiency and physical and mental health in a longitudinal survey of welfare recipients. *Journal of Health and Social Behavior*.

[7] Dewing S., Tomlinson M., Le Roux I. M., Chopra M., Tsai A. C. (2013). Food insecurity and its association with co-occurring postnatal depression, hazardous drinking, and suicidality among women in peri-urban South Africa. *Journal of Affective Disorders*.

[8] Motbainor A., Worku A., Kumie A. (2016). Level and determinants of food insecurity in East and West Gojjam zones of Amhara Region, Ethiopia: a community based comparative cross-sectional study. *BMC Public Health*.

[9] McLaughlin K. A., Green J. G., Alegría M. (2012). Food insecurity and mental disorders in a national sample of U.S. adolescents. *Journal of the American Academy of Child & Adolescent Psychiatry*.

[10] Siefert K., Heflin C. M., Corcoran M. E., Williams D. R. (2001). Food insufficiency and the physical and mental health of low-income women. *Journal of Women and Health*.

[11] Hassan B. K., Werneck G. L., Hasselmann M. H. (2016). Maternal mental health and nutritional status of six-month-old infants. *Revista de Saúde Pública*.

[12] Jebena M. G., Taha M., Nakajima M. (2015). Household food insecurity and mental distress among pregnant women in Southwestern Ethiopia: a cross sectional study design. *BMC Pregnancy and Childbirth*.

[13] Ali D., Saha K. K., Nguyen P. H. (2013). Household food insecurity is associated with higher child undernutrition in Bangladesh, Ethiopia, and Vietnam, but the effect is not mediated by child dietary diversity. *Journal of Nutrition*.

[14] Hadley C., Patil C. L. (2006). Food insecurity in rural Tanzania is associated with maternal anxiety and depression. *American Journal of Human Biology*.

[15] Tefera T. B., Erena A. N., Kuti K. A., Hussen M. A. (2015). Perinatal depression and associated factors among reproductive aged group women at Goba and Robe Town of Bale Zone, Oromia Region, South East Ethiopia. *Maternal Health, Neonatology and Perinatology*.

[16] Garcia J., Hromi-Fiedler A., Mazur R. E. (2013). Persistent household food insecurity, HIV, and maternal stress in Peri-Urban Ghana. *BMC Public Health*.

[17] Ethiopian Federal Ministry of Health. (2014). *Ethiopia’s Fifth National Health Accounts*.

[18] Coates J., Swindale A., Bilinsky P. Household Feed Insecurity Access Scale (HFIAS) for Measurement fo Food Access: Indicator Guide.

[19] Suesenberg M., Orley J. (1994). *A users guide to the slf reporting qustionnaire (SRQ)*.

[20] Kortmann F. (1987). Problems in communication in transcultural psychiatry. The serlf-reporting questionnaire in Ethiopia. *Acta Psychiatrica Scandinavica*.

[21] Kortmann F. (1988). Comprehension and motivation in responses to a psychiatric screening instrument validity of the SRQ in ethiopia. *The British Journal of Psychiatry*.

[22] Tafari S., Aboud F. E., Larson C. P. (1991). Determinants of mental illness in a rural Ethiopian adult population. *Social Science & Medicine*.

